# How experiencing eudaimonic emotions through music shapes prosocial and altruistic behavior: introducing a Unified Model of Music-Listening-Induced Eudaimonia (UMMIE)

**DOI:** 10.3389/fcogn.2025.1705976

**Published:** 2025-11-28

**Authors:** Rebecca Tukachinsky Forster, Daniel H. Spitz, Tal-Chen Rabinowitch, Roni Granot

**Affiliations:** 1 Chapman University School of Communication, Orange, CA, United States; 2 Robert Efroymson Music Cognition Lab, The Hebrew University of Jerusalem, Jerusalem, Israel; 3 School of Creative Arts Therapies, University of Haifa, Haifa, Israel

**Keywords:** music listening, prosocial behavior, eudaimonic emotions, being moved, transcendence, awe, power ballad, altruism

## Abstract

This paper introduces the Unified Model of Music-Listening Induced Eudaimonia (UMMIE), which offers an integrated novel theoretical framework to explain how music listening promotes prosocial and altruistic outcomes. Drawing on communication and psychology research, UMMIE maintains that elicitation of eudaimonic emotions can be appraised inwards, towards oneself, and thus contribute to the individual's personal growth, wellbeing, and development, or outwards, by connecting to something bigger than oneself, to humanity in general, which could possibly then lead to enhanced prosocial and altruistic attitudes and behaviors. The model specifies musical, situational, and individual variables that serve as moderators and increase the likelihood of experiencing inwards or outwards effects of music listening. In this way, the current article organizes and synthesizes existing literature and offers a novel blueprint for future research.

## Introduction

Ample research has demonstrated the potential of listening to music to promote prosocial outcomes (e.g., Wu et al., 2025). For example, studies found that listening to music can increase prosocial decision-making (McDonald et al., 2022) and charitable behavior (Hong et al., 2023, 2025); promotes intercultural understanding (Clarke et al., 2015); and reduces prejudice (e.g., Miranda and Gaudreau, 2020); and enhances empathy toward outgroups (Bodner and Bergman, 2017) as well as enhance human fellow feelings (Ansani et al., 2024).

Various mechanisms have been proposed to explain these effects, including, for example, synchronization (for a review, see Pardo-Olmos et al., 2025) and intergroup contact (e.g., Bodner and Bergman, 2017). However, despite the growing evidence for the role of music listening in promoting social functioning and fostering a more harmonious society, this literature is dispersed, and there is a need for a comprehensive model integrating the various factors and mechanisms underlying this process. This manuscript presents a theoretical model Unified Model of Music-Listening-Induced Eudaimonia (UMMIE), that explicates the effects of music listening on prosocial and altruistic behavior, specifically through inducing eudaimonic emotions.

Several theoretical models (Janicke-Bowles, 2025; Oliver et al., 2021) have been proposed to theorize how consuming narratives (i.e., reading or watching films) that elicit eudaimonic experiences can lead to prosocial and altruistic outcomes. While some parallels could be drawn from these models to music listening, their heavy reliance on plot and character components of the stimuli preclude a simple mapping to the ways music may attain these effects.

**Figure 1:**
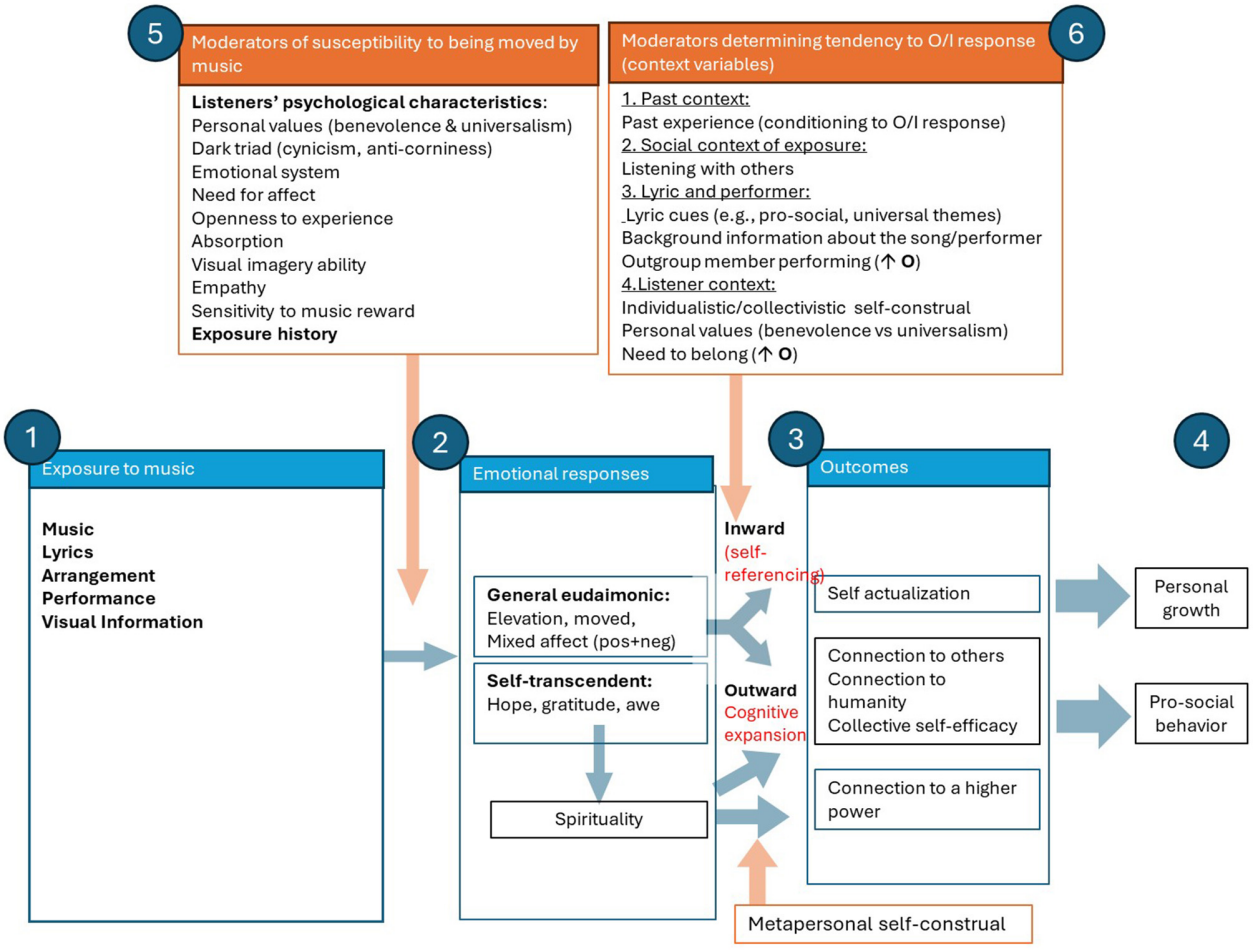
Unified Model of Music-Listening Induced Eudaimonia (UMMIE) Model of the pathways from music listening to eudaimonic outcomes. Boxes 1 and 2 focus on the key characteristics of the music (1) and the type of eudaimonic emotions it may elicit (2). Boxes 3 and 4 show how these may lead through cognitive appraisal to inward (personal growth) and outward (prosocial attitudes and behaviors) outcomes. Box 5 specifies listener's psychological characteristics and past history that may moderate their susceptibility to experience eudaimonic emotions, and box 6 lists various variables that may shape the cognitive appraisal that leads to an inward vs. an outward effect as indicated by the arrow with ‘O' or ‘I'.

The current manuscript updates, extends, and fits these efforts to music listening (as opposed to active music production) and proposes a comprehensive model that articulates the emotional and cognitive mechanisms that mediate the eudaimonic effects of listening to music on prosocial and altruistic outcomes (see Figure 1). It delineates key characteristics of music (box 1), the associated emotional and cognitive processes that mediate these effects (boxes 2–3), and outlines the boundary conditions of these effects by identifying their contextual and individual-level moderators (boxes 5–6). The following sections start with describing a generalized model of the effect of eudaimonic experience on prosocial and altruistic outcomes (box 4), followed by an application of the model to the experience of listening to music.

## Eudaimonic emotions

Aristotle maintained that the highest form of happiness is that of self-fulfillment by living a life of virtue and purpose (Huta and Ryan, 2010). Eudaimonia stands in contrast to hedonia, which focuses on short-term enjoyment, pleasure, and comfort derived from maximizing pleasure and minimizing distress (e.g., sadness), for instance, by pursuing fun activities that offer escapism (Moyer-Gusé and Wilson, 2024). Conversely, eudaimonia constitutes a long-term flourishing by living a life of meaning, the pursuit of excellence, growth, and authenticity (Huta and Ryan, 2010). More specifically, eudaimonia can be achieved through the fulfillment of specific core universal needs, as articulated by self-determination theory (Martela and Ryan, 2020; Ryan and Martela, 2016): competence (sense of mastery and accomplishment), relatedness (mutually caring relationships), and autonomy (sense of self-direction and ownership of one's behavior and thoughts).

In contrast to hedonic enjoyment, eudaimonic enjoyment is not incompatible with negative affect, as long as it carries meaning. A well-known example is the pleasure people derive from tragic art, often referred to as the “paradox of tragedy” (Knobloch-Westerwick et al., 2013). According to Aristotle, this paradox is resolved through *catharsis*—a process of emotional purification that restores psychological balance and moral understanding by allowing audiences to experience and release negative emotions (Scheele and DuBois, 2013). Eudaimonic enjoyment is fostered by the experience of poignancy, or mixed affect (i.e., simultaneously feeling both positive and negative emotions). Eudaimonic affective responses also include several complex emotions, such as elevation, appreciation, tenderness, being touched, and being moved (Oliver et al., 2012). Additionally, eudaimonia encompasses self-transcendent emotions such as gratitude and awe. Self-transcendence refers to states of consciousness characterized by a decreased focus on the self, accompanied by feelings of connectedness, awe, and moral elevation (Yaden et al., 2017). These eudaimonic feelings are accompanied by physiological responses, such as the feeling of warmth in the chest, a lump in the throat, tears, chills, and goosebumps (Oliver et al., 2012).

### Elicitors of eudaimonic responses

Eudaimonic responses are deeply personal, based on selective perception and interpretation processes, situated within one's personal experiences, personality, and situational factors. For example, on average, individuals are likely to experience drama and tearjerkers as eudaimonic and view comedy as hedonic. Yet, a sizable minority of individuals report finding deeper meaning in comedy and feel elevation when watching what others might regard as shallow entertainment (Janicke and Oliver, 2015; Oliver et al., 2012). Similarly, in the context of music, eudaimonic responses to music are highly individual. In fact, (Konečni 2005, p. 37) suggested that “the associative web that is interposed between the sound and the state of being touched is unique to each person” and precludes any generalizations.

Despite these idiosyncrasies, research has identified factors that elicit eudaimonic experiences. In other words, eudaimonia is not restricted to any particular genre, and although some genres are nominated more than others [e.g., more documentaries than other genres and romance than comedies (Raney et al., 2018)], these differences are rooted in the specific characteristics of the message that are more likely to produce effects.

(Janicke-Bowles 2025) distinguishes between ***modeled*** and ***direct elicitors*** of eudaimonic responses. The former involves a character portraying eudaimonic emotions such as hope, gratitude, tenderness, and love, allowing the viewer to co-experience these emotions vicariously through identification with that character. Second, certain themes and message characteristics can trigger eudaimonic responses without mediating the character's eudaimonic expressions. For instance, depictions of the vastness and grandness of nature trigger awe, while witnessing others' expressions of love and connection (birth, reunion) lead to feelings of Kama muta (“being moved by love”). The insights from research on narratives are also relevant to music, with the need to identify both specific direct musical triggers of eudaimonia and more general eudaimonia-provoking themes.

## Pathways of effects of eudaimonic emotions

Once elicited, eudaimonic responses can trigger cognitive elaboration that lies along a continuum ranging from inward-directed to outward-directed cognitions (Oliver et al., 2018). On one end of the spectrum, the feelings of elevation and tenderness can lead to self-reflection, as individuals dwell on their personal experiences, memories, aspirations, and place in life. Such inward-facing elaboration is aligned with the motivation for self-authenticity or autonomy (in terms of the self-determination theory). As such, it fosters personal growth through better actualization of one's own image of the self (Oliver et al., 2012). Conversely, mixed affect and self-transcendent emotions can promote outward-directed elaboration. This appraisal encompasses cognitive expansion and connection to something beyond oneself, leading to self-actualization through needs for connectedness with others (Oliver et al., 2012; Janicke-Bowles, 2025).

### Prosocial and altruistic outcomes of the outward pathway of eudaimonic emotions

The path of outward elaboration has been theorized to lead to prosocial and altruistic behavior through several underlying mechanisms. First, self-concept is a dynamic construct that can be attenuated by environmental stimuli, including music. Outward elaboration on eudaimonic experiences activates the interdependent self-construal (prioritizing group and relationships over autonomy and personal goals) and metapersonal aspects of the self (connecting to the universe and higher power), thereby guiding individuals to act on the goals and motivations of others (Janicke-Bowles, 2025). Thus, eudaimonic emotions, such as gratitude, Kama muta, and awe, that may expand one's identification beyond the individual self, lead to helping, cooperation, integration into social groups, and inclusive intergroup attitudes (e.g., Bai et al., 2017; Piff et al., 2015; Seo et al., 2023). Second, the sense of connectedness not only fosters empathy but also prompts individuals to act on these prosocial feelings. Isolation leads to feeling powerless and seeing one's actions as meaningless, whereas a sense of interconnectedness boosts collective efficacy and empowers individuals to take action (Moyer-Gusé and Wilson, 2024). Third, self-transcendent messages that trigger spirituality alongside interconnectedness further compel individuals to strive for unity with others and the universe and to act on their morality (Oliver et al., 2018; Janicke-Bowles, 2025).

In line with this theorization, studies have consistently found a link between exposure to stimuli that evoke eudaimonic emotions and various prosocial and altruistic outcomes. Experimental literature consistently shows a link between experiencing awe and a feeling of “small self,” which in turn scales up to global/human identification and broader moral concern, generosity, helping, cooperation, forgiveness, and moral expansiveness (Liao et al., 2024; Piff et al., 2015; Song et al., 2023; Wu et al., 2024; Zining and Mahmud, 2023). There is further indication that awe heightens connectedness/oneness, sometimes feeding empathy, producing downstream prosocial outcomes and stigma reduction (Luo et al., 2022, 2023), increases global citizenship identification (i.e., universalism) and shifts donations toward global (vs. local) causes (Seo et al., 2023).

Revisiting an awe-provoking experience reduced aggression after playing a violent video game (Yang et al., 2016) and increased participants' readiness to volunteer their time to help others (Rudd et al., 2012). Watching awe-eliciting videos increased generosity across different contexts (Piff et al., 2015); increased motivation to help and actual helping behavior (Aquino et al., 2011; Schnall et al., 2010; Schnall and Roper, 2012); and hindered stereotyping (Krämer et al., 2017). Similarly, watching elevating videos increased prosocial motivation (Erickson et al., 2018) and feelings of connectedness with members of an out-group racial group (Oliver et al., 2015). Furthermore, exposure to videos that elicit overall eudaimonic emotions was found to lead to greater motivation for morality, altruism, helping others (Chen et al., 2023; Oliver et al., 2012; Ott et al., 2021), and reduced stigmatization (Bartsch et al., 2016).

The model presented above represents the literature on eudaimonic emotion that has been primarily studied and empirically tested in the context of various audio-visual narratives, such as movies. However, the model applies to the context of music (both instrumental and songs). The following sections will synthesize the literature in psychology of music to articulate the possible pathways of effects of listening to music on altruistic and prosocial behaviors through music-induced eudaimonic experiences and identify the boundary conditions of these effects. We begin by conceptualizing the relationship between exposure to music (box 1) and experiencing eudaimonia (box 2).

## UMMIE: Unified Model of Music-Listening Induced Eudaimonia

### Listening to music eliciting eudaimonic responses

Music has been identified as a central source for eudaimonic emotions. According to a national survey in the U.S., listening to music was the most common source of feeling moved, touched, or inspired (90.5%), ahead of watching movies and television shows or reading news stories and books (Raney et al., 2018). In fact, seeking eudaimonia was theorized as one of the core motivations for music consumption (Groarke and Hogan, 2020).

(De Leeuw et al. 2022) found that music that participants identified as meaningful (vs. pleasurable) elicited, even in a laboratory setting, greater eudaimonic emotions (being-moved, seeking meaningful themes in life, and a desire to express love), although both types of music elicited intense mixed emotions and a feeling of connectedness. Despite the importance of these results, little can be concluded about what made the specific musical choice meaningful or pleasurable, and if or how these very personal choices could be generalized (as will be discussed later in the overview of Boxes 1 and 5 in the model).

### Outcomes of music-elicited eudaimonia

As stated earlier, eudaimonic experiences can be appraised outwards (connection to others) or inwards (self-referencing) leading to different outcomes. Indeed, research on music found evidence of both pathways. Although these studies did not specifically measure eudaimonic mediators, this research found that listening to music has inward effects on agency (Saarikallio et al., 2020); self-empowerment (Elvers et al., 2018); self-identity (DeNora, 1999) and purposefulness (Lamont, 2011). Outward effects include social bonding and connection (Gabrielsson, 2011; Joseph and Southcott, 2014; Miranda and Gaudreau, 2011; Perkins and Williamon, 2014). Eudaimonic responses also fostered empathy and compassion and increased immediate prosocial decisions (McDonald et al., 2022). Preferred chill-inducing music has been linked to greater dictator-game allocations in small samples without mechanism tests (Fukui and Toyoshima, 2014). It is important to note that music listening has also been shown to have an association with negative social aspects, such as increased aggression and decreased prosocial behavior, specifically in adolescents who listen to music with aggressive lyrics (Coyne and Padilla-Walker, 2015). Similar accounts have also been demonstrated in adults listeners in the works of Greitemeyer and others (Fischer and Greitemeyer, 2006; Warburton et al., 2024) and have been reviewed recently (Barlett and Kjaervik, 2025; Olsen et al., 2023). It is apparent that listening to aggressive-oriented music might in effect, result in more aggression and in reduced prosocial behaviors. Due to the negative nature of the lyrics, an open question remains, as to whether such negative emotions could lead to eudaimonia, which requires a combination of both positive and negative emotions at its core. With this in mind, it seems to us that listening to aggressive-oriented music do not gets the listener past Box 1, and so there is probably no direct or in-direct way onto Box 2, where the experience of eudaimonia occurs. Of course, future experimental work could take aggressive-oriented music listening as a specific case study for our UMMIE model.

Thus far, we have established the general path between music exposure and eudaimonia (the link between boxes 1, 2, 3 and 4 in the model). However, there are still many open questions regarding what music elicits eudaimonia, and who, and under what circumstances, is most likely to be affected by it? Moreover, what determines whether the eudaimonic response is channeled toward helping others (through the outward elaboration path) or is leveraged to promote self-growth through the inward elaboration path?

We now turn to identifying the key variables that lead to eliciting eudaimonic responses to music and articulating the situational and psychological moderators of these effects (box 5 in the model). Next, we will turn to theorizing the variables that can contribute to the impact of music-elicited eudaimonic emotions and identifying the variables responsible for diverting the elaboration process inwards or outwards (box 6 in the model), ultimately leading to prosocial and altruistic behaviors.

### Music elicitors of eudaimonic emotions

As seen in Figure 1, we differentiate between two types of music-induced eudaimonic emotional responses (box 2) that could mediate the various outcomes: General eudaimonic emotional responses of elevation and *being-moved*, and self-transcendence emotions of *awe and wonder*. While the former have been associated with feelings of connectedness, mediated by empathy (De Leeuw et al., 2022; Eerola et al., 2016; Swarbrick and Vuoskoski, 2023; Vuoskoski et al., 2022), the later seem to be associated with mixed emotions of fear and wonder, induced by vastness, and expanded or loss of sense of time and space leading to feelings of humility, spirituality and wanting to merge with others, with nature or a higher power (Becker, 2004; Burcke, 1757/2007; Gabrielsson, 2011; Konečni, 2005; Levinson, 2024; Rouget, 1985). As we will discuss below, they are elicited in different contexts, through different types of music, and are influenced by a large set of possible moderators.

#### Elicitation of Being Moved

Most of the seminal studies that examined being moved by music focused on uses and responses to sad music (Peltola and Eerola, 2016; Vuoskoski and Eerola, 2017; Garrido and Schubert, 2011). They found that responses to such music were associated with mixed emotions carrying a sense of nostalgia and personal memories (Eerola and Peltola, 2016; Van den Tol et al., 2016), pleasure and concomitantly activation of the reward system (Eerola et al., 2021), wonder and a sense of meaning—primarily through personally meaningful lyrics (Brattico et al., 2011) and a sense of beauty (Vuoskoski et al., 2011). (Vuoskoski et al. 2022) attempted to define the acoustic and musical characteristics that contribute to the feeling of being moved, but were only partially successful in doing so since only a limited set of features showed consistent results, mostly related directly or indirectly to intensity.

The being-moved emotion induced by music is often accompanied by the same physiological responses reported by (Oliver et al. 2012) of warmth in the chest, a lump in the throat, tears, chills, and goosebumps. The musical features associated with chills or frisson (Harrison and Loui, 2014; Panksepp, 1995; Salimpoor et al., 2011) include various types of musical surprises (sudden harmonic changes, sudden changes in dynamics, sudden entrance of a solo voice), crescendi, appoggiaturas, and high-pitched vocal solos (Bannister, 2020; de Fleurian and Pearce, 2021; Huron, 2006; Sloboda, 1991).

We postulate that music (with its specific arrangement, performance, and visual information) that includes eudaimonic topics as displayed in its lyrics (if existent); and is capable of eliciting similar physiological responses associated with being-moved as described above; and/or embodies some aspects of the phenomenology of the non-aesthetic emotion of being moved as expounded for example in (Cova and Deonna 2014) and (Menninghaus et al. 2015), may be more prone to elicit the being-moved feeling. Building on Cova and Deonna's conceptualization, we stress that it may not be enough to have a sense of mixed joy-sadness emotions. Rather, there has to be some “emergence of the positive from the negative”, like a moral triumph following some struggle (Cova and Deonna, 2014, p. 451). Following Meninghaus et al. we maintain that the feeling qualities of being moved are associated with wide, warm, large, soft, round, and uplifting rather than their contrasts. These last can be partially mapped to musical qualities through cross-modal associations where loudness and slow tempo are acoustic features relevant to size (loud and slow = big), legato articulation is associated with roundness (Eitan, 2017), a gradual crescendo could be associated with wideness, and the right choice of instruments and registers can bring in the feeling of warmth (Rosi et al., 2022).

None of these elements on its own is a prerequisite (e.g., even unfamiliar instrumental music presumably devoid of explicit “core-values” topics can be moving—see e.g., Vuoskoski et al., 2022), or a sufficient condition (one may experience chills but not be moved by the music), but their combination enhances the probability of obtaining the effect. Specifically, we assume that some combination of such characteristics could more readily elicit mechanisms of empathic listening and fantasy engagement, contagion, and associative conditioning (Juslin, 2013), whereby previous encounters of similar music in artistic or narrative (e.g., peak emotional moments in films) and social contexts (e.g., peak emotional moments in a wedding), may all converge to create a feeling of being moved.

### The power ballad as a possible prototype for a moving song

Rather than focusing on specific musical elements, we suggest looking for music genres whose main aim is to move their listeners by combining the elements mentioned above, such as the “*Power Ballad*”. Some might say that this kind of music or songs uses unabashedly all formulas and clichés for obtaining this precise goal with great success (see Table 1).

**Table 1:** Power ballad characteristics relevant to the emotion of being moved.

**Characteristics**	**Realization**
Lyrics: eudaimonic themes	Love, loss, farewell, death
Sentimentality: bigger than life emotional expressive display	A display of the pain and sorrow that gives way to mixed emotions. The sorrow portion at the beginning of the ballad is typically (1) In slow-mid tempo (2) Introduced by a soft accompaniment of a single acoustic instrument (3) With very light drum beat and bass (4) Soft or even vulnerable voice
Mixed emotions through the “Uplifting effect” that colors the pain with other emotional colors	Hope, triumph, joy, euphoria mainly through the constant intensification of the music: (1) Escalating intensity each time to a higher plateau through additional background voices, additional instruments including large orchestral sound especially strings (tremolando, pulsating) (2) Increase in emphatic beat (3) Vocal performance of increased volume, local upward glissandi, out-cries, and powerful but audibly strained rise to highest peaks of pitch and intensity
Formulaic in its process = opportunity for surprise	Typically, one or even two upward harmonic shifts by a tone creating (1) Higher tension due to harmonic abrupt change and higher pitched melody (2) Release due to fulfillment of expectation Ample of chances for performer to introduce extra-long pauses

The power ballad occupies a unique space between Epic music and a traditional Ballad. A traditional ballad is defined by its storytelling nature, often lyrical, simple, and focused on conveying a narrative in a straightforward manner. Ballads are frequently melancholic, telling stories of loss, tragedy, or unfulfilled love, and they often end on a sad or unresolved note. Epic music, by contrast, emphasizes scale, grandeur, and atmosphere, often evoking vast landscapes, heroic struggles, or cosmic dimensions, making it a staple in soundtracks and orchestral works. It celebrates magnitude and triumph, often portraying overwhelming victories or a sense of monumental achievement.

Unlike the tragic endings of many traditional ballads or the cosmic victories of epic music, power ballads dramatize personal struggles—self-doubt, heartbreak, longing—and resolve them in smaller yet deeply meaningful victories. In this way, the power ballad bridges personal sentiment with musical intensity, making private emotion feel monumental without losing its everyday resonance. As (Metzer 2012, 2016) clarifies, the power ballad is a song in a mid-to slow tempo (definitely not fast in order to distinguish it from the “up-tempo dance music”), dealing mainly with feelings of *love and loss*, and is characterized by what he calls “sentimentality and uplifting”. Topics of sentimentality often overlap those of being moved since they touch on lost love and painful farewell in life and death. The uplifting feeling is embodied in the song's constant rise in sonic and emotional energy providing the “positive out of the negative” effect of hope or resilience to endure the loss.

Importantly, this general outline can be realized in any genre—country, rock, R &B, or crossovers with classical music (e.g., “Il Divo”), and each genre will define its vocal style, instrumentation, and performance venues. Indeed, the power ballad is found in films (Celine Dion—“My Heart Will Go On”), musicals (e.g., in Disney's “The Beauty and the Beast” or “Mulan”), video games, and TV talent reality show (Metzer, 2012).

The characteristics of the power ballad relevant to feelings of being moved are presented in Table 1.

Though the power ballad is a genre of popular music, classical instrumental music, especially from the romantic period, has its own examples of “power pieces”—some of which have become prime examples of sad and moving music like Samuel Barber's *Adagio for strings*. Examining the examples from (Vuoskoski et al. 2022), Smetana's *Vlatava* is clearly based on the same scheme, as is also, *Morning Mood* by Grieg, though in a somewhat more lyrical tone.

In summary, the power ballad offers in its lyrics, vocal rendition, and stock of expressive formulas all the components that have the potential to induce the being moved emotion: It touches on core attachment experiences, it has mixed emotions with the “positive emerging from the negative”, or uplifting sentimentality, and it has many features associated with chills such as crescendi, high pitched tones, sudden harmonic shifts, drum rolls, and more.

#### Music and Self-Transcendence

As stated earlier, self-transcendence has been conceptualized both as a trait and as a state that combines reduced self-centeredness with a sense of connectedness to others or to a larger power (Peterson and Seligman, 2004). (Yaden et al. 2017) suggest viewing it as a transient state “involving the dissolution of boundaries between the sense of self and ‘other”' (p. 143). Researchers have identified several varieties of Self-Transcendence Experiences (STEs), some of which are closely linked to music: Flow (often in the context of music performance; Groarke and Hogan, 2020); feelings of awe or the sublime (Bonner and Friedman, 2011; Keltner and Haidt, 2003); peak experiences (Gabrielsson, 2011; Lamont, 2011); and religious, spiritual, or mystical experiences, sometimes accompanied by altered states of consciousness (Becker, 2004; Rouget, 1985).

In order to understand the role of music in these experiences it is useful to focus on two underlying subcomponents postulated by (Yaden et al. 2017): The reduction of the self to the point of dissolution of its bodily boundaries, involving possibly a loss of or reconfigured sense of space and time, and a sense of connectedness or merging with other people, nature, or a higher power.

An anecdotal example of this experience can be found in Gabrielsson's “Strong Experiences with Music: Music is Much More than Just Music” (2011, section 13.3A), as a woman recounts:

“[the cantor] played for me Bach's Toccata and Fugue in C major on the organ. I sat there in the gloom in the empty church hall and it felt as if my heart would burst because I was in such raptures. I myself and the church hall expanded in some way and merged in a larger context—a part of the universe, perhaps.”

#### Music and Awe or the Sublime through Vastness

There is very little empirical data on music and the specific feeling of awe or sublime (Konečni, 2005, 2008; Zentner et al., 2008), as a separate category from being-moved or chill-inducing. The 18th-century philosopher Edmund Burke associated the sublime with a feeling similar to terror but one that does not carry with it real danger, hence brings with it pleasure (1757/1958, p. 54). He suggested that in comparison to the beautiful, it should be vast, massive, rugged, dark, even gloomy, yet the two—the beautiful and the sublime can blend in art (pp. 238–239).

Awe-inducing vastness may arise from perceptual experiences (e.g., the Grand Canyon, an endless sky) or from vast soundscapes such as a large orchestra, a massed choir, or an organ resonating in a cathedral (Konečni, 2005). Electronic effects like reverb, echo, and panning can simulate this acoustic vastness even in smaller spaces.

Awe can also stem from conceptual vastness—from encompassing theories to intricately complex artworks (Keltner and Haidt, 2003). The two types of vastness may lead to a sense of wonder, joy, and a sense of a “small self” (Levinson, 2024; Piff et al., 2015; Shiota et al., 2007), that, as described above, can scale into global/human identification and prosocial attitudes and behaviors (although see (Villar et al. 2022), for a “darker” possible outcome of willingness to sacrifice the individual for the global good). (Keltner and Haidt 2003) argue that vastness requires cognitive accommodation [echoing Kant's view of the sublime, as cited in (Levinson 2024)], creating opportunities for shifts in perspective. In its extreme, this accommodation can take the form of altered states of consciousness as described below.

Examples of music inducing the sublime offered in the musicological literature include pieces that combine religious/biblical stories with large performing forces (orchestra and choir) such as the “Creation” by Haydn, the big oratorios by Handel (Hibberd and Stanyon, 2020), as well as purely instrumental music such as Arvo Pärt's, “Tabula Rasa”, 2nd movement *Silentium*, the *Andante tranquillo* from the first movement or Bartok's “Music for Strings, Percussion and Celesta” with its expansive contrapuntal intricacies or the *Adagio* from Bruckner's 8th symphony (Levinson, 2024). Levinson also identifies “negatively transcendent” works that have an engulfing but terrifying power such as Penderezcki's “Threnody for the Victims of Hiroshima”, that according to our UMMIE model might create withdrawal and an inward self-referential effect.

Taken together, these examples suggest that awe-inspiring music often shares features such as religious or cosmic themes, vast soundscapes, slow tempi with possible long stretches of bass drones, complex textures that defy a sense of clear units or boundaries, and defiance of regular structures—without overwhelming tension.

#### Music and Spiritual or Mystical Experiences

Clearly, all music associated with religious or spiritual contexts has the potential to elicit feelings of transcendence. Moreover, when accompanying religious or secular rituals, music serves as an organizing force and as an emotional glue that fosters collective effervescence (Durkheim, 1912/1976) and communal synchrony (Fiske et al., 2019). Some researchers even propose that music and dance evolved as cultural mechanisms for inducing altered states of consciousness (ASC) that are required for strengthening social bonds (Freeman, 2000; Tarr et al., 2014).

Indeed, ASC are often cultivated through music in shamanic rituals, where sounds represent spirits, communicate with them, and assist in creating sensory overload through increasing rhythmic and motoric intensity (Rouget, 1985; Becker, 2004).

In a very different context, ASC were central to the countercultural movements of the 1960s, where psychedelic music—often combined with drug use—sought to expand consciousness and explore new sensory experiences of self and its relation to the universe (Leary, 1968/1998).

More recently, in rave and house music culture, music combined with psychedelics and dance has been reported to elicit mystical experiences, sensory exploration, and feelings of connection to others and to nature (Mosina and Michael, 2024). Finally, certain music (both electronic and orchestral) associated with “space music”, such as Ligeti's ethereal “Lux Aeterna” (famously used in Kubrick's, 1968: “A Space Odyssey”), can suggest cosmic vastness and extraterrestrial contact (Weinel, 2018; Wierzbicki, 2014), demonstrating another genre in which music contributes to shaping spiritual and mystical experiences.

Despite the large variance of sonic manifestations of music associated with mystical and spiritual experiences, one might suggest that beyond the context, the main characteristic of such music its attempt mimic auditory experiences, that dissolve the boundaries between fantasy and reality especially in the domains of time and space.

### Moderators of susceptibility to eudaimonic responses to music

As stated earlier, while some music can be innately more “eudaimonic” than other music, even where research identified specific direct elicitors, there is a relatively large individual variability in how particular listeners respond to it (e.g., in the context of chill-inducing music: Bannister, 2020; Grewe et al., 2009; Panksepp, 1995; Salimpoor et al., 2011).

Thus, it is critical to consider the individual characteristics and contextual variables that increase or decrease the likelihood of certain musical qualities to induce eudaimonic responses. It is thus possible that the exact same music can elicit profound awe, self-transcendence, and elevation in one person, can be experienced as boring or even irritating by another. The following section identifies a variety of potential moderators of exposure to music and eudaimonic responses. The moderators (box 5) are organized in three general categories: Lyrics, individual differences (e.g., personality and value systems), and exposure history. We also describe these variables, their associated predictions and measures in Appendix A.

### Lyrics

The proposed model distinguishes between the effects of the *music* and the *lyric* arguing that while the music itself can elicit eudaimonic emotions, this effect can be amplified when the music is coupled with verbal messages (i.e., lyrics) that include eudaimonic themes. Lyrics can include both direct and modeled elicitors of eudaimonic feelings.

An analysis of stimuli used to elicit eudaimonic experiences in experimental research identified the common themes across eudaimonic stories (de Graaf and Das, 2023). These included a combination of a threat to needs for safety or relatedness and a virtue of humanity, love, or kindness. Similarly, (Watts 2022) in her interviews with parents identified death or injury and human connection as the two leading themes in eudaimonic movies that their children consume. It is, therefore, logical to assume that in the context of music listening, lyrics (in songs, musicals, or opera) that depict these themes will similarly be more likely to elicit eudaimonic responses. For example, (Greitemeyer 2009) found that listening to songs with prosocial lyrics, compared to neutral ones, increased prosocial thoughts, empathy, and helping behavior. Eudaimonia-eliciting lyrics blend positive and negative tones, engaging with simultaneously relatable and larger-than-life themes, such as love, loss, death, hope, and despair. Power ballads often exemplify this approach, employing lyrics that navigate the tension between vulnerability and resilience, struggle and hope, pain and the possibility of triumph.

### Moderators determining tendency for inwards and outwards eudaimonic responses

Now that we have discussed the factors that facilitate eudaimonic responses, the question is, when would the response be channeled inwards (personal growth) vs. outwards, resulting in greater altruistic and prosocial outcomes? The following sections identify moderating variables (Box 6) that are hypothesized to direct listeners' cognitions either toward self-referencing or toward cognitive expansion to include others and connect to humanity at large. The proposed UMMIE model roughly divides these variables into several circles of context, related to the music and the listener.

### Exposure history

In part, differences in an individual's susceptibility to experience eudaimonic emotions through music listening may stem from personal experiences that serve as a perceptual lens that attenuates or amplifies individuals' responses to messages. For example, research on eudaimonia in the context of narratives found that when stories resonate with the readers' experiences (e.g., loss and bereavement), the message is experienced as more profound (e.g., Das and Peters, 2022; Koopman, 2013). It is not implausible that a message component (a word, imagery) that is completely benign for some individuals will carry a special personal meaning for a particular individual, triggering self-referencing or outward elaboration, thereby shifting that person's experience to a more eudaimonic one. Moreover, contextual factors can bias a person's responses to the message, making the same individual likely to respond differently to the eudaimonic message depending on their pre-existing affective state and cognitive accessibility (e.g., Das and te Hennepe, 2022). Music, too, can elicit stronger eudaimonic responses if it resonates with the listeners' experiences and prior emotional states. Individuals can find deeper meaning in lyrics that relate to their experiences or activate schemas (Semino, 1995).

Furthermore, the meaning of the music can be amplified over the course of repeated exposure through learning and growing familiarity (Gold et al., 2019; Schoeller et al., 2025; Szpunar et al., 2004). “Mere exposure” work shows that liking tends to rise with repetition, but only up to a point, and that people would prefer music with a balance of predictability and surprise (Gold et al., 2019). However, over longer exposure, the chance of feeling chills drops, but the chills people do report can feel more intense, suggesting fewer but sharper peaks with repetition (Schoeller et al., 2025). Taken together, it seems that repetition teaches listeners what to expect, shifts preferences toward moderately complex pieces, makes peak moments rarer but sometimes more powerful, and strengthens the role of familiarity and specific structural events in shaping enjoyment and being moved by music. Such repetition teaches listeners what to expect, shifts preferences toward moderately complex pieces, makes peak moments rarer but sometimes more powerful, and strengthens the role of familiarity and specific structural events in shaping enjoyment and being moved by music.

### Lyrics cues and background information

The lyrics themselves and background information about the song can prime individuals to appraise the elicited eudaimonic emotions either to a private or social route. For example, some lyrics specifically focus on personal plights (e.g., *November Rain*) while others include explicit references to societal issues, making explicit calls for connectivity and expanding oneself to others (*We are the World, Lean on Me*). Other lyrics may prime ingroup themes that enhance group cohesion and distinctiveness (e.g., anthems, sports chants), sometimes simply by being written in a language associated with a particular group.

Thus, the lyrics themselves invite individuals to either retrieve personal memories and process the music in a self-referential manner or through cognitive expansion. However, this distinction is not straightforward. Many lyrics are open-ended and polysemic, allowing individuals to insert different meanings—social or personal into them, triggering both cognitive processes. For example, descriptions of situations, memories, or emotions connected to the listener's own experiences may induce nostalgia, as autobiographical salience is the strongest predictor of music-evoked nostalgia (Sterenberg Mahon and Roth, 2023). Such lyrics guide listeners inward, encouraging self-referencing, reflection, and a focus on close personal relationships rather than broader societal concerns. As such, it fosters self-continuity and reflection and strengthens bonds with close others, situating the eudaimonic experience primarily within the personal and relational domain rather than extending outward to universal compassion.

However, nostalgia can be directed toward specific people, objects, or situations, as well as toward collective experiences, such as longing for the “good old days” or for a place that no longer exists. Future research should examine whether nostalgia focused on different targets, personal vs. communal, affects the direction of eudaimonic emotions evoked by music, and how such emotions may be oriented toward ingroups or outgroups and subsequently translated into prosocial behavior.

Moreover, even socially centered lyrics employ symbolic metaphors to articulate emotions that are otherwise difficult to describe, providing both a means of connection for listeners and an additional layer of aesthetic value, but also making it more open to interpretation. In such cases, knowledge of the background behind the song can attenuate how it is interpreted. For example, in Les Misérables, the “I Dreamed a Dream” can be interpreted within the context of the *Les Misérables* musical, as addressing questions of social injustice, freedom, and the struggle of good against evil in society. However, when listening to the song performed by Susan Boyle in the 2009 audition for Britain's *Got Talent*, the song takes on another meaning. Performed by an underdog contestant who defies stereotypes and overcame personal challenges to reach this platform, the song becomes a symbol of personal triumph and perseverance. In other words, the same music and lyrics can be primed to make different core eudaimonic themes (outward–connectedness vs. inwards–competency and autonomy).

#### The Performer's Identity

The identity of the performer as a member of the listener's outgroup (vs. ingroup) can also amplify the outward potential of the eudaimonic music. Several studies have shown that exposure to music can constitute an intergroup contact that reduces prejudice toward outgroup members (e.g., Bodner and Bergman, 2017; Harwood et al., 2016). Conceivably, the outgroup status of a performer of eudaimonic music makes the social (rather than personal) more salient and direct the listeners' eudaimonic responses to the music down the society-oriented, rather than inward-facing pathway.

### Personal experiences and history

Songs or melodies taught from an early age, from parent to child, can foster both a sense of belonging and personal attachment. The mere act of listening to or singing such songs may evoke a motivation to help others within one's ingroup, as individuals develop strong emotional connections to these people through their own memories, family members, and friends (“episodic memory” mechanism in Juslin, 2013). Other lyrics prime ingroup themes that enhance group cohesion and distinctiveness (e.g., anthems, sports chants), sometimes simply by being written in a language associated with a particular group. Lyrics taught from an early age, from parent to child, can foster both a sense of belonging and personal attachment. Other music has always been experienced in specific contexts in a way that conditions collective-minded responses (e.g., religious music or national music such as ‘the bugle call *Taps'*).

### Listeners' psychological characteristics

A second class of moderators includes individual psychological differences that predispose some individuals to be more moved and transcendent. These include values, traits, and other neuropsychological differences.

#### Values

One core individual difference that may play a significant role in how individuals respond to music is a person's value system. Schwartz's theory of fundamental human values offers a comprehensive framework, identifying ten competing motivational domains organized in a circular structure, which has been validated across 82 countries (Schwartz, 2012). Some of these values, particularly benevolence (caring for one's close others) and universalism (concern for humanity and nature more broadly), should, in theory, make a listener more likely to experience eudaimonia when listening to music. Conversely, those who prioritize values associated with self-enhancement (hedonism, achievement, and power) tend to appreciate self-transcendence values less and are less expected to act on such motivations.

#### Openness

Openness to experience can be seen as a gateway to eudaimonia, as it predisposes individuals to embrace moments of awe, wonder, and profound engagement that transcend ordinary aesthetic pleasure (Silvia et al., 2015). Individuals high in this trait are more inclined to expand their thinking and feeling boundaries, turning encounters with nature, art, or music into meaningful experiences that foster personal growth and a more profound sense of uniqueness. Thereby, openness functions not only as a personality trait but as an essentially aesthetic disposition that promotes moments of existential significance (Silvia et al., 2015). Through its association with heightened sensitivity to frisson and aesthetic chills in music (Colver and El-Alayli, 2016), and with preferences for complex or intense musical genres that encourage reflection and emotional exploration (Vella and Mills, 2017), openness supports a fuller integration of cognitive and emotional dimensions of musical experience.

#### Absorption

Absorption reflects a state of complete immersion, often accompanied by a temporary loss of self-consciousness or altered perception of time and space. It represents a complex cognitive state involving both focused attention and mind wandering (Høffding et al., 2024) and encompasses multiple ways in which individuals can become deeply engaged with music (Vroegh, 2019). (Palhares et al. 2024) hypothesized that absorption plays a central role in how music alters mental states, while (Herbert 2016) proposed that it functions as a self-regulatory process, enabling trance- or hypnotic-like experiences. Individuals who experience absorption during music listening may be particularly susceptible to eudaimonic experiences, as they can transcend everyday experiences and use music as a medium to access meaningful emotional states that might otherwise be difficult to achieve.

#### Dark Triad

The “Dark Triad” is a constellation of three conceptually distinct but empirically overlapping personality traits: Machiavellianism, Narcissism, and Psychopathy. Machiavellianism refers to a strategic, manipulative orientation toward others and a strong focus on self-interest. Narcissism is characterized by an inflated sense of self-importance and entitlement. Psychopathy involves impulsivity, callousness, and a lack of remorse or empathy (Furnham et al., 2013). Notably, although all values in their core are positive and important motivations, Machiavellianism and Narcissism tend to align with the values of Achievement and Power, whereas Psychopathy is more closely linked with Hedonism and Power values (Kajonius et al., 2015).

Unsurprisingly, individuals who score high on dark triad personality traits perceive eudaimonic stories as inauthentic and corny (Appel et al., 2019). Although self-reported feelings of being touched, moved, and inspired in response to eudaimonic videos were largely unaffected by the dark triad, the corniness responses translated to a more negative overall evaluation of the eudaimonic content. By extension, it is possible that the dark triad acts as a buffer against the eudaimonic potential of music. Some music genres, such as power ballads, use a narrative structure featuring positivity that grows out of the negativity. Also, people with Machiavellianism, narcissism, and psychopathy might be more prone to treat such music as a cliché, shmaltz, or even fake rather than an authentic, powerful, and uplifting experience.

#### Disposition for Emotional Experiences

Affective neuroscience theory posits that emotions arise from evolutionarily conserved neural systems, emphasizing the biological foundations of affective experience and their role in shaping cognition and behavior (Panksepp, 2004). We assume that experiencing eudaimonia through music requires integration of multiple affective systems. Specifically, the *seeking* (exploration and motivation) and *care* nurturing and attachment) systems are crucial, as they underlie curiosity about others, the establishment of social bonds, and the maintenance of interpersonal connections. Together, these systems foster a sense of social connectedness. Moreover, the *panic/grief* (separation distress) system contributes emotional recognition of social and emotional pain, thereby supporting empathy. Integration of these systems is, therefore, proposed to create readiness for eudaimonic experiences in response to music. Indeed, (Laeng et al. 2016) found that activation of the *rage* (anger and aggression) system reduces the occurrence of musical chills. When survival-oriented systems such as *rage* dominate, attentional resources may be constrained, limiting the potential for eudaimonic engagement with music.

Relatedly, Need For Affect (NFA) refers to an individual's tendency to approach or avoid emotional experiences (Maio and Esses, 2001). Those high in NFA actively seek emotionally rich situations, such as music, and engage deeply with them, facilitating reflection, empathy, and personal growth—core aspects of eudaimonic experiences. In contrast, low-NFA individuals tend to avoid intense emotions, potentially limiting their access to music's transformative and meaningful effects. Indeed, studies on eudaimonic experiences in other forms of entertainment found a negative association between NFA and eudaimonic enjoyment (Raney et al., 2018). Thus, since NFA serves as the core motivation for avoiding or approaching emotional stimuli (Maio and Esses, 2001; Appel et al., 2019; Leone and Presaghi, 2007), it is logical to assume that NSA shapes how deeply and personally people experience the eudaimonic benefits of music.

#### Trait Empathy

Empathy influences the inherent potential and capacity of individuals to experience eudaimonic emotions when listening to music. Across lab experiments, online studies, and concert surveys, a clear pattern emerges: individuals who score higher on affective empathy—especially empathic concern and a tendency to experience others' feelings—report feeling more moved by music, most reliably with sad or tender pieces (Eerola et al., 2016; Swarbrick et al., 2021; Taruffi and Koelsch, 2014; Vuoskoski et al., 2022; Vuoskoski and Eerola, 2017). Empathic concern specifically predicted higher overall and peak feelings of being moved, while fantasy-like imagination showed smaller, context-dependent links (Vuoskoski et al., 2022). As for chills, openness to experience—and, in large samples, absorption—consistently predict chills occurrence and intensity during music listening (Colver and El-Alayli, 2016; Nusbaum and Silvia, 2011; Schoeller et al., 2025). Empathic concern also predicts greater spontaneous body movement when listening to music (Zelechowska et al., 2020). In children, fantasy and perspective-taking predicted stronger emotions and liking for sad music (Kawakami and Katahira, 2015). These findings are consistent with research on overall eudaimonic experiences. Surveys consistently demonstrate that eudaimonic enjoyment is positively associated with trait empathy, and in particular, with empathetic concern (Choi and Lapierre, 2025; Raney et al., 2018).

Overall, it seems that the capacity for empathy plays an important role in an individual's susceptibility to be moved by music and to have, in the first place, a stronger predisposition to experience kama muta.

#### Music-evoked Visual Imagery

Across studies, music-evoked visual imagery (MEVI)—internally generated visual scenes, movements, and colors during music listening—is frequent in adult listeners and exhibits structured content that is partly stimulus-dependent and partly idiosyncratic (Borgohain et al., 2023; Dahl et al., 2023; Hashim et al., 2023; Küssner and Eerola, 2019). MEVI relates to affective engagement: imagery vividness and positive imagery valence predict higher aesthetic appeal and liking (Belfi, 2019), and concurrent self-reports of visual imagery covary with global felt emotional intensity during listening (Hashim et al., 2023). Research establishes MEVI as a relatively stable individual characteristic that, although common, varies across individuals, creating a pattern of person-specific “signatures” (Dahl et al., 2023; Hashim et al., 2023). Accordingly, MEVI could drive musical listening toward more pleasurable, aesthetic and higher emotional intensity experiences. However, not all individuals would be prone to experience MEVI at all and MEVI could also be experienced in different frequencies and intensities, making MEVI a central moderator of susceptibility of being moved by music.

#### Sensitivity to Musical Rewards

Musical reward refers to the individual differences in the degree to which people experience music as pleasurable, a phenomenon closely linked to the neural activity of the reward system. Specifically, the dorsal and ventral striatum release dopamine in response to pleasurable music, coding the reward value of musical excerpts (Ferreri et al., 2019). (Mas-Herrero et al. 2012) conceptualized musical reward as comprising five components: Musical Seeking (the tendency to explore and listen to music), Sensory-Motor (dancing and moving to music), Mood Regulation (using music to modulate affective states), Emotional Evocation (actively experiencing and seeking emotions in music), and Social Reward (sharing and bonding with others through music). Individuals who derive pleasure from multiple aspects of music may be more likely to experience eudaimonic or meaningful emotions, as their engagement with music facilitates richer emotional and social interactions. In particular, the emotional and social dimensions of musical reward can foster shared musical experiences that create lasting memories, generating a feedback loop in which meaningful engagement further motivates exploration and social musical participation. Although musical reward generally declines with age (Belfi et al., 2022), younger listeners may be particularly susceptible to these eudaimonic experiences. These processes also relate to absorption, as individuals highly responsive to musical reward are more likely to become fully immersed in music, experiencing intense focus and deep emotional engagement.

### Social context of listening

Listening to the same music in a social context (rather than as a solitary activity) can also promote more outward eudaimonic response. Specifically, appraisal of the eudaimonic feelings as outwards rather than inwards can be attenuated by a sense of synchronization experienced when individuals are listening to music together.

Across studies of music listening and participation, social context reliably shapes how people experience music. *Live audiences* tend to feel more connected to other audience members and experience greater connectedness than livestream viewers (Swarbrick and Vuoskoski, 2023). On the same vein, online collaborative playlist work suggests that simply *believing a human partner was present* can engage self–other processes, especially in younger participants (Harris and Cross, 2021). In participatory contexts, *synchrony* was found to be a robust driver of social orientation: dancing in time increases ratings of interaction quality and partner-directed looking (Bamford et al., 2023), and synchronized group dance elevated social closeness and pain thresholds relative to partial or asynchronous movement (Tarr et al., 2014). In children, synchronous movement seems to enhance closeness and perceived similarity (Rabinowitch and Knafo-Noam, 2015), boost cooperative behavior (Rabinowitch et al., 2024; Rabinowitch and Meltzoff, 2017), reduce interpersonal distance (Tunçgenç and Cohen, 2016), and, when paired with music, increase sharing compared with visual-only synchrony, suggesting a music-specific boost to prosociality (Cross et al., 2024). Remarkably, this effect is evident even in early development: 14-month-old infants who were bounced in synchrony with an experimenter were subsequently more likely to help the experimenter compared to those bounced asynchronously (Cirelli et al., 2014).

In mixed-reality ensembles, adding music and increasing partner realism enhanced shared agency and self–other merging (Van Kerrebroeck et al., 2023). Overall, it seems that co-presence, synchrony, and interaction affordances increase connectedness, shared agency, and prosocial behavior in musical settings. Synchrony also extends beyond dyads to group contexts. (Reddish et al. 2014, 2016) showed that synchronous movement not only increased prosociality toward fellow performers but also generalized to outsiders, including members of different groups. Taken together, these findings suggest that listening to music together with others should shift the focus of the individual from inward (which possibly serves as the “baseline” or “default” option), to an outward state, where they are open to more readily connect to others, to humanity and possibly also to a higher power than oneself.

### Outer-inner orientation

Another important determinant of whether eudaimonic emotions will be directed inward (toward the self and close relationships) or outward (toward humanity more broadly) relates to their general tendency for self vs. other orientation. Individuals with a stronger interdependent self-construal may be predisposed to experience music as a way of connecting with others and spending time together, making them more susceptible to the inward path of eudaimonic experience. For such listeners, music becomes tied to shared memories and relationships. By contrast, individuals with a strong independent self-construal may also benefit from the inward path, but in a different way—focusing less on interpersonal connections and more on personal growth, self-reflection, and identity exploration through music.

Beyond the independent and interdependent orientations, (DeCicco and Stroink 2007) proposed a third dimension: the metapersonal self-construal. While still related to the other two, the metapersonal self represents a spiritual self in which the boundary between the individual and the environment dissolves, creating a sense of unity with all things. In this state, objects and entities once considered external to the self, merge with it. This self-construal is distinct from the independent and interdependent forms and often emerges from cultural, religious, or spiritual traditions. (Stroink and DeCicco 2011) found that each self-construal is associated with different value orientations: interdependence with benevolence (caring for close others), independence with self-direction (personal growth and autonomy), and metapersonal with spirituality and universalism. A strong metapersonal self-construal may therefore increase susceptibility to outward eudaimonic experiences, such as compassion, awe, and concern for humanity and the environment. Supporting this, prior research has shown that metapersonal self-construal is linked to greater emotional wellbeing (Mara et al., 2010), cooperation, and environmental concern (Arnocky et al., 2007). Thus, music that elicits eudaimonic responses can take listeners through different paths (inward/outward) depending on the listeners' self-construal.

## Conclusions and future directions

This manuscript applies the rich body of communication and psychology research on audio-visual to music listening. Importantly, it is not possible for the complex phenomenon of eduaimonia to be reduced to a prescriptive formula. Rather, the proposed UMMIE model identifies variables, including musical elements, situational factors, and individual characteristics that may increase the likelihood of eliciting these experiences, offering testable hypotheses that can be empirically examined using a variety of methods, including experimental manipulation of music/lyric characteristics and individual differences (e.g., priming to increase salience of different self-construals); contextual variables (e.g., co-listening, background information). This comprehensive model also identifies areas that have been understudied, offering a blueprint for a research agenda, as outlined below.

### Understanding variations in music experiences

Numerous songs develop similarly, increasing arc or energy. What, then, makes some pieces more moving than others? Using the variables identified in the UMMIE model, future research can begin unpacking some of these questions: Is it the intensity at which the peak arrives? The specific pace at which escalation develops? The brevity or number of moments of tension release? The vocal rendition? How important is the presence of a human voice in this type of emotion? As for the feeling of vastness in music (also formulated as one aspect of a loss of sense of space), it has not been studied at all. What other types of emotions does vastness create? Can it create a sense of pure joy? Sadness? What is the effect of acoustic vastness as compared to conceptual vastness? Assuming it is in some way related to space perception, can we study this association empirically?

### Understanding the Interplay of Lyrics and Music

The focus of UMMIE is on music. However, drawing on theories of narrative communication, UMMIE acknowledges the potential of lyrics to amplify these effects. However, texts of songs are more akin to poetry than to narrative and do not employ the same set of narrative devices as stories or films do. Future research should examine more closely the types of messages conveyed in music, how they differ from those narratives, and why musical messages may be particularly effective in evoking eudaimonic emotions (Levy et al., 2024). One aspect to consider is the slow unfolding of music compared to text, a temporal broadening often extended by instrumental “replied” or sections. giving the listener ample time to immerse themselves in visual imagery, memories, and cognitive appraisals.

### Additional Moderators

The model lists key moderators that make some listeners more susceptible to music's eudaimonic effects. However, additional variables can be considered in the future, even though the current evidence is not robust enough to include them in the model. For example, the amount of musical training has been shown to influence neural structure (Barrett et al., 2013) and, therefore, may shape how individuals respond to or analyze music (Martínez-Castilla et al., 2021). Although musical training is not always directly linked with music-induced pleasure (Cheung et al., 2019), it may, nonetheless, play an important role within different stages of the UMMIE model and should be examined systematically. Additionally, the subjective importance of music in an individual's life has been identified as a critical factor in shaping emotional responses (Granot et al., 2021), making it another key variable to integrate into future applications of the model.

### Cultural Differences

Future studies should examine whether our model applies cross-culturally, as research on music psychology addresses both universal and culturally specific aspects of music. In what ways can it relate to non-Western philosophies that treated music as a means for fostering better cosmic and human harmony, mutual understanding, and morality enhancement through aesthetic transcendence? Also of interest is whether globalization has created a universal musical “language” that resonates across cultures in order to transmit narratives common to humanity. Power ballads, for instance, appear in diverse contexts worldwide, whether rooted in human emotional universals or shaped by cultural globalization. Even in collectivist and tight Eastern cultures such as Japan, power ballads follow a similar emotional arc, moving from despair toward hope.

## Data Availability

The original contributions presented in the study are included in the article/Supplementary material, further inquiries can be directed to the corresponding author.
