# Supplementary material for: How experiencing eudaimonic emotions through music shapes prosocial and altruistic behavior: introducing a Unified Model of Music-Listening-Induced Eudaimonia (UMMIE)
Source: Front Cognit. 2025 Nov 28;4:1705976. doi: 10.3389/fcogn.2025.1705976 (PMC13281109; doi:10.3389/fcogn.2025.1705976)
Supplement: Supplementary file 1 [file Data_Sheet_1.pdf]

Appendix A

List of Moderators, Mediators and Their Measures

Variable	Prediction of the effect	Measures
**Moderators of the effects of exposure on eudaimonic responses**		
Listener's psychological characteristics:		
Personal values	Listeners who score higher on benevolence and universalism will experience greater eudaimonic responses	Schwartz Value Survey and the Portrait Values Questionnaire (Schwartz, 2012)
Dark triad	Listeners who score higher on Machiavellianism, and psychopathy will experience lower eudaimonic responses	Short Dark Triad (SD-3) (Jones & Paulhus, 2014)
Emotional system	Listeners who score higher on seeking, care, and panic/grief will experience greater eudaimonic responses	Affective Neuroscience Personality Scales (Davis & Panksepp, 2011)
Need for affect	Listeners who score higher on need for affect will experience greater eudaimonic responses	Need for Affect Questionnaire (NAQ-S) (Appel et al., 2012)
Openness	Listeners who score higher on openness to new experiences will report greater eudaimonic responses	The Big Five Inventory (BFI) (John et al., 1991)
Absorption	Listeners who score higher on trait absorption will experience greater eudaimonic responses	Modi®ed Tellegen Absorption scale (MODTAS) (Jamieson, 2005)
Visual imagery	Listeners who have a higher tendency to produce music-evoked visual imagery experience greater eudaimonic responses	Self-report and behavioral measures (Gelding et al., 2022)
Empathy	Listeners who score higher on trait empathy will experience greater eudaimonic responses	Interpersonal Reactivity Index (IRI) (Davis, 1983)
Sensitivity to music reward	Listeners who score higher on sensitivity to music reward will experience greater eudaimonic responses	Barcelona Musical Reward Questionnaire (BMRQ) (Mas-Herrero et al., 2013)
Lyrics		
Eudaimonic cues in lyrics	Lyrics with eudaimonic themes will increase the eudaimonic effect of music	Coding scheme for themes pertaining to acts of kindness, moral virtue, death/injury (deGraaf & Das, 2023; Watts, 2022)
Exposure History		

Repeated exposure	Repeated exposure will increase the eudaimonic effect of music	Asking about past exposure or experimental manipulation of repeated exposure.
**Moderators predicting eudaimonic inward vs outward pathway**		
Past Experience		
Past experience	Having a relevant personal experience will increase self-referencing (i.e., follow the inward pathway), resulting in greater personal growth.	Asking about relevant experience (e.g., Das & Peters, 2022)
Social Context		
Exposure alone vs with others	Listening with others will increase cognitive expansion and connection to others/humanity (i.e., follow the outward pathway), resulting in greater altruism/prosocial behavior.	Asking about the context of exposure or experimental manipulation of the social context of exposure.
Lyric and Performer		
Lyric theme	Lyrics containing prosocial and universal themes will increase cognitive expansion and connection to others/humanity (i.e., follow the outward pathway), resulting in greater altruism/prosocial behavior.	Coding scheme capturing the presence of general themes (“us”, “people”).
Background story	Background information about the music being written in response to a personal struggle will increase self-referencing (i.e., follow the inward pathway), resulting in greater personal growth.<br>Background information about the music being written in response to a bigger social struggle will increase cognitive expansion and connection to others/humanity (i.e., follow the outward pathway), resulting in greater altruism/prosocial behavior.	Coding scheme capturing personal vs. societal cues (personal struggle vs collective struggle).
Performer’s group membership	When music is performed by an outgroup member, the listener will experience greater cognitive expansion and connection to others/humanity (i.e., follow the outward pathway), resulting in greater altruism/prosocial behavior.	Asking about the performer’s group membership or experimental manipulation of the (alleged) group membership of the performer to match/mismatch the listener’s group identity.
Listener’s context		
Self-construal	Listeners with greater collectivistic and metapersonal self-construal will experience greater cognitive expansion and connection to others/humanity	Self-Construal Scale (SCS) (Singelis, 1994)

	(i.e., follow the outward pathway), resulting in greater altruism/prosocial behavior.<br>Listeners with greater individualistic self-construal will experience greater self-referencing (i.e., follow the inward pathway), resulting in greater personal growth.	The Metapersonal Self Scale (MPS) (DeCicco & Stroink, 2007)
Personal values	Listeners who score higher on benevolence and universalism will experience greater cognitive expansion and connection to others/humanity (i.e., follow the outward pathway), resulting in greater altruism/prosocial behavior.	Schwartz Value Survey and the Portrait Values Questionnaire (Schwartz, 2012)
Need to belong	Listeners who score higher on need to belong will experience greater cognitive expansion and connection to others/humanity (i.e., follow the outward pathway), resulting in greater altruism/prosocial behavior.	Need to Belong Scale (NTBS) (Leary, 2013)
**Eudaimonic responses (mediators of the effect of music on prosocial/altruistic outcomes)**		
General Eudaimonic:		
Emotions	Experiencing greater elevation and being moved increases eudaimonic outcomes	Appreciation (Oliver & Bartsch, 2010)
Mixed affect	Experiencing both positive and negative affect increases eudaimonic outcome	Emotional range (Ott et al., 2021)
Self-transcendence	Experiencing greater hope, gratitude, and awe increases eudaimonic outcomes	List of discrete emotions and a transcendence elevation scale (Janicke-Bowles et al., 2022)
Inward-path		
Self-reflection	Experiencing greater self-referencing, thinking about oneself, increases self-growth	Self-referencing (DeGraaf, 20214)<br>Self-related thoughts (Janicke-Bowles et al., 2022)
Outward-path		
Cognitive expansion	Experiencing greater connection to others, humanity, and higher power increases altruistic/prosocial outcomes	Connectedness to humankind (Oliver et al., 2015)<br>Connectedness with others and higher power (Janicke & Oliver, 2017)

References

Appel, M., Gnambs, T., & Maio, G. R. (2012). A Short Measure of the Need for Affect. *Journal of Personality Assessment*, *94*(4), 418–426. https://doi.org/10.1080/00223891.2012.666921

Das, E., & Peters, J. (2022). “They never really leave us”: Transcendent narratives about loss resonate with the experience of severe grief. *Human Communication Research*, *48*(2), 320-345.

Davis, K. L., & Panksepp, J. (2011). The brain's emotional foundations of human personality and the Affective Neuroscience Personality Scales. *Neuroscience & Biobehavioral Reviews*, *35*(9), 1946-1958.

Davis, M. H. (1983). Measuring individual differences in empathy: Evidence for a multidimensional approach. *Journal of Personality and Social Psychology, 44*(1), 113–126. https://doi.org/10.1037/0022-3514.44.1.113

De Graaf, A. (2014). The effectiveness of adaptation of the protagonist in narrative impact: Similarity influences health beliefs through self-referencing. *Human Communication Research*, *40*(1), 73-

DeCicco, T. L., & Stroink, M. L. (2007). A third model of self-construal: The metapersonal self. International Journal of Transpersonal Studies, 26, 82-104.. *International Journal of Transpersonal Studies, 26* (1). https://doi.org/10.24972/ijts.2007.26.1.82

Gelding, R. W., Day, R. A., & Thompson, W. F. (2022). Music-evoked imagery and imagery for music: Subjective and behavioural measures. In *Music and mental imagery* (pp. 77-87). Routledge.

Jamieson, G. A. (2005). The modified Tellegen absorption scale: A clearer window on the structure and meaning of absorption. *Australian Journal of Clinical and Experimental Hypnosis*, *33*(2), 119-139.

Janicke, S. H., & Oliver, M. B. (2017). The relationship between elevation, connectedness, and compassionate love in meaningful films. *Psychology of Popular Media Culture*, *6*(3), 274.

Janicke-Bowles, S. H., Jenkins, B., O'Neill, B., Thomason, L., & Psomas, E. (2022). Other-focus versus self-focus: The power of self-transcendent TV shows. Psychology of Popular Media, 13(1), 34-43. Advance online publication. https://doi.org/10.1037/ppm0000441

Ott, J. M., Tan, N. Q. P., & Slater, M. D. (2021). Eudaimonic Media in Lived Experience: Retrospective Responses to Eudaimonic vs. Non-Eudaimonic Films. *Mass Communication and Society*, *24*(5), 725–747. https://doi.org/10.1080/15205436.2021.1912774

John, O. P., Donahue, E. M., & Kentle, R. L. (1991). *Big Five Inventory (BFI)* [Database record]. APA PsycTests. https://doi.org/10.1037/t07550-000

Jones, D. N., & Paulhus, D. L. (2014). Introducing the short dark triad (SD3) a brief measure of dark personality traits. *Assessment*, *21*(1), 28-41.

Leary, M. R. (2013). *Need to Belong Scale (NTBS)* [Database record]. APA PsycTests. https://doi.org/10.1037/t27154-000

Mas-Herrero, E., Marco-Pallares, J., Lorenzo-Seva, U., Zatorre, R. J., & Rodriguez-Fornells, A. (2013). *Barcelona Music Reward Questionnaire (BMRQ)* [Database record]. APA PsycTests. https://doi.org/10.1037/t31533-000

Oliver, M. B., Kim, K., Hoewe, J., Chung, M. Y., Ash, E., Woolley, J. K., & Shade, D. D. (2015). Media-induced elevation as a means of enhancing feelings of intergroup connectedness. *Journal of Social Issues*, *71*(1), 106-122.

Schwartz, S. H. (2012). An overview of the Schwartz theory of basic values. *Online Readings in Psychology and Culture, 2*(1). https://doi.org/10.9707/2307-0919.1116

Singelis, T. M. (1994). The measurement of independent and interdependent self-construals. *Personality and Social Psychology, 20(*5), 580-591 https://doi.org/10.1177/0146167294205014
